# Prescription Department

**Published:** 1895-04

**Authors:** 


BRONCHO-PNEUMONIA IN CHILDREN

Dr. Frank S. Parsons, of Northampton, Mass., in the Journal of the American Medical Association, gives the following formula :—

℞ Vini ipecac...... ʒj.

Potass. citratis... ʒss.

Tinct. opii camph, ʒij.

Elixir simplicis.. ℥j.

Aquæ destill......q. s. ad ℥iv.

M. Sig.: A teaspoonful every two hours to an infant 1 month old.

LARYNGEAL IRRITATION.

℞ Alcohol (40°)............ʒv.
Menthol..................gr. viij.
Cocain. hydrochlorat....gr. ij.
Acid. benzoic ...........gr. xv.

M. Sig.: Use as a gargle or spray. Add 10 or 20 drops to half a glassful of warm, borated water.—*La Riforma Medica*.

139. BILIOUS COLIC.

℞ Spir. chlorof........2.4 (ʒss)

Fl. ext. dioscorea visc..0.4(grs. vi)

Spir. vini. rect.........4.0 (ʒj)

Glycerine and water qu. s. ad...30.0 (℥j)

4cc. (ʒj) every half or one to six hours, according to indication.

CYSTITIS.

℞ Acid. benzoici..........ʒiij.

Sodii botat.,............ʒij.

Aquæ..................℥vj.

M Sig.: Tablespoonful three times a day.

—*Prof. J. V. Shoemaker.*

LEAD COLIC.—(*Dr. A. R. Loomis.*)

℞ Opii....................gr. i.

Ext. belladon............gr. 1-6.

Ol. croton................gtts. i.

M. Sig.: Every two hours, until relief is obtained.

ACUTE CATARRHAL PHARYNGITIS.

Pencil the throat several times a day with an hydroalcoholic solution of cocaine (the formula of which has been given), or once or twice daily, with tannic acid :—

℞ Acid. tannic......gr. xv. xxx.

Glycerin.......... ʒiiss.—M.

CHRONIC PHARYNGITIS.

In the dry form pencil the throat with

℞ Acid lactic ........ ʒi¼.

Aq. destillat....... ʒvj.—M.

Or,

℞ Salol ............ gr. xv.

Ol. vaselin... .... ℥ss.—M.

Or,

A-naphthol,

Pulv. camphor ...aa gr. xxiv.

Ol. vaselin......... ℥ss.—M.

TOOTHACHE.

Dr. Ben. H. Broadnax, of Broadnax, La., packs the cavity with cotton moistened in a mixture thus composed :—

Rub together equal parts of
Carbolic acid (liq.)
Gum-camphor,
Chloral hydrate.
Menthol,
Glycerin.—*Jour. Mat. Med.*

SCIATICA.

Dr. Metcalf orders :—

℞ Tinct. aconit.
Tinct. colch. sem.
Tinct. bellad.
Tinct. actæa racem., aa equal parts.

M. Sig. : Six drops every six hours.
—*Medical and Surgical Reporter.*

